# Identification, Structural and Functional Characterization of Dormancy Regulator Genes in Apricot (*Prunus armeniaca* L.)

**DOI:** 10.3389/fpls.2019.00402

**Published:** 2019-04-05

**Authors:** Eszter Balogh, Júlia Halász, Alexandra Soltész, Zsolt Erös-Honti, Ádám Gutermuth, László Szalay, Mária Höhn, Attila Vágújfalvi, Gábor Galiba, Attila Hegedüs

**Affiliations:** ^1^Department of Genetics and Plant Breeding, Faculty of Horticultural Science, Szent István University, Budapest, Hungary; ^2^Department of Plant Molecular Biology, Agricultural Institute, Centre for Agricultural Research, Hungarian Academy of Sciences, Martonvásár, Hungary; ^3^Department of Botany and Soroksár Botanical Garden, Faculty of Horticultural Science, Szent István University, Budapest, Hungary; ^4^Department of Pomology, Faculty of Horticultural Science, Szent István University, Budapest, Hungary; ^5^Festetics Doctoral School, Georgikon Faculty, University of Pannonia, Keszthely, Hungary

**Keywords:** apricot, *CBF*, *DAM*, dormancy, flower development, gene expression, microsporogenesis

## Abstract

In the present study, we identified and characterized the apricot (*Prunus armeniaca* L.) homologs of three dormancy-related genes, namely the *ParCBF1* (*C-repeat binding factor*), *ParDAM5* (*dormancy-associated MADS-BOX*) and *ParDAM6* genes. All highly conserved structural motifs and the 3D model of the DNA-binding domain indicate an unimpaired DNA-binding ability of *Par*CBF1. A phylogenetic analysis showed that *ParCBF1* was most likely homologous to *Prunus mume* and *Prunus dulcis CBF1*. *Par*DAM5 also contained all characteristic domains of the type II (MIKC^C^) subfamily of MADS-box transcription factors. The homology modeling of protein domains and a phylogenetic analysis of *ParDAM5* suggest its functional integrity. The amino acid positions or small motifs that are diagnostic characteristics of DAM5 and DAM6 were determined. For *ParDAM6*, only a small part of the cDNA was sequenced, which was sufficient for the quantification of gene expression. The expression of *ParCBF1* showed close association with decreasing ambient temperatures in autumn and winter. The expression levels of *ParDAM5* and *ParDAM6* changed according to *CBF1* expression rates and the fulfillment of cultivar chilling requirements (CR). The concomitant decrease of gene expression with endodormancy release is consistent with a role of *ParDAM5* and *ParDAM6* genes in dormancy induction and maintenance. Cultivars with higher CR and delayed flowering time showed higher expression levels of *ParDAM5* and *ParDAM6* toward the end of endodormancy. Differences in the timing of anther developmental stages between early- and late-flowering cultivars and two dormant seasons confirmed the genetically and environmentally controlled mechanisms of dormancy release in apricot generative buds. These results support that the newly identified apricot gene homologs have a crucial role in dormancy-associated physiological mechanisms.

## Introduction

Many important fruit tree species belong to the *Prunus* genus of the *Rosaceae* family. Flower bud development is one of the most critical stages in their reliable production. Spring frost injury is the most common reason for yield-loss in producing countries at the Northern Hemisphere under temperate climate (Kaya et al., 2018). The annual growing cycle of temperate woody plants forms an integrated system with subsequent phases of active growth and dormancy (Perry, 1971; Lloret et al., 2018). During dormancy plants exhibit little or no growth and their metabolic activity decreases for a period of time. It is an essential strategy for perennial plants to survive harmful environmental conditions during winter (Hanninen and Tanino, 2011). Dormancy can be divided into two phases, the endo- and ecodormancy (Lloret et al., 2018). Endodormancy is a genetically controlled mechanism that is triggered in early autumn by external factors and inhibits bud development even under growth-promoting conditions. Plants require a certain amount of chill for endodormancy-release to enter ecodormancy phase when bud growth is only prevented by unfavorable climatic conditions.

The alternation of phenological stages in *Prunus* is chiefly regulated by the ambient air temperature (Welling and Palva, 2006; Heide, 2008). During autumn, low temperature induces dormancy; after dormancy break during winter warming in spring causes bud burst and the rapid development of flower organs. Plants give different responses to the seasonal change of environmental conditions, depending on species or even cultivar/eco-type (Hanninen and Tanino, 2011). In case of *Prunus* species, chilling-accumulation during winter plays a key role in the induction of budbreak and flowering (Ruiz et al., 2007; Alburquerque et al., 2008). Over dormancy progression, characteristic histological and physiological alterations occur in flower buds. The anther developmental stages (archesporium, string, pollen mother cell, tetrad and pollen stages) are most often distinguished to follow microsporogenesis (Szalay et al., 2019). Dormancy is considered to mark a boundary between the development of the sporogenous tissue in the anther and the occurrence of pollen meiosis (Julian et al., 2011).

The genetic factors responsible for the cold- and frost stress responses first have been identified in *Arabidopsis thaliana* (Baker et al., 1994). The CBF transcription factors belong to the DREB1 subfamily within the AP2/ERF protein family. They are able to bind to the CRT/DRE (C-repeat/dehydration responsive element) sequence motif in the promoter region of the regulated gene, which contains a conserved CCGAC sequence as a binding site for the DNA-binding domain of CBF proteins (Stockinger et al., 1997; Liu et al., 1998). On low temperature, the *Arabidopsis CBF* genes can be induced within 15 min and in 2 h they activate the “CBF-regulon,” i.e., the cold-regulated genes which contain the CRT/DRE regulatory-element (Yang et al., 2005).

In the *Rosaceae* family, *DREB/CBF*-type genes have been first described in sweet-cherry (*Prunus avium* L.) and their amino acid sequences were approximately 50% identical to the *Arabidopsis CBF*s (Kitashiba et al., 2002; Owens et al., 2002). The expression of one sweet-cherry *CBF* homolog (*CGI-B*) caused increased frost resistance in transgenic *Arabidopsis* lines (Kitashiba et al., 2004). A full-length cDNA of a peach *CBF* gene (*PpCBF1*) has been isolated by Wisniewski et al. (2011) and its ectopic expression in apple resulted in induced dormancy and increased cold hardiness. The *CBF* gene sequences of apple and peach seemed to be very similar, especially concerning their AP2 binding domain (Wisniewski et al., 2011). In almond (*Prunus dulcis* Mill.), two *CBF* sequences were identified and their transcription level was in correlation with the expression of a *dehydrin* gene, *PdDHN1* (Barros et al., 2012b). Dehydrins are encoded by a group of genes related to abiotic stress response, such as frost or drought in many species. *CBF* genes were identified in many other plant genera, such as *Vitis* (Xiao et al., 2006), *Populus* (Benedict et al., 2006), *Betula* (Welling and Palva, 2008), *Eucalyptus* (Gamboa et al., 2007) and *Triticum* (Galiba et al., 2009; Soltész et al., 2013).

Previous studies on *Arabidopsis*, snapdragon (*Antirrhinum majus* L.) and petunia hybrids shed light on the role of *MADS-BOX* genes in controlling flower-organogenesis (Theissen et al., 2000; Horvath, 2009). Among *Rosaceae* species, the role of *MADS-BOX* genes in bud dormancy was first proved in the “*evergrowing*” (*evg*) peach mutant that is incapable of forming terminal vegetative buds in response to dormancy-inducing conditions. Bielenberg et al. (2008) made a comparative mapping to a 132-kb region of the wild-type genome where six genes were found to be missing from mutant plant tissues. These six *MADS-BOX*-type genes form a cluster in the wild-type genome, while there is a 41,746-bp deletion in the same region of the *evg* genome. This six *DAM* (*DORMANCY-ASSOCIATED MADS-BOX*) genes are strong candidates for the regulation of growth cessation and dormancy induction in peach. *PpDAM*s were suggested to be orthologs of the *Arabidopsis SHORT VEGETATIVE PHASE* (*SVP*) and *AGAMOUS-LIKE 24* (*AGL24*) (Yamane et al., 2011a), two genes that are involved in flowering (Gregis et al., 2009).

The expression of *DAM* genes seems to be tissue-specific, moreover, each of them has a clear seasonal expression-pattern presumably regulated by photoperiod (Li et al., 2009). The seasonal expression level of *DAM5-6* genes shows correlation with bud dormancy induction and break in case of peach, *P. persica* L. (Yamane et al., 2011a,b). Previous studies revealed that both peach and apple *DAM*-like genes contain a conserved element in their promoter region that is highly homologous to the consensus *CBF*-binding sites (Wisniewski et al., 2011; Mimida et al., 2015). The temporal arrest of floral organ enlargement is presumably controlled by *PmDAM5-6* (Jiménez et al., 2010; Yamane et al., 2011a). Specific period of low temperature exposure is necessary to reduce the expression of *PmDAM5-6* and to break this inhibition; therefore, they possibly work as dose-dependent growth inhibitors in dormant buds (Yamane et al., 2011a,b). Several *DAM*-like genes have been found in other *Rosaceae* species such as Japanese apricot (*Prunus mume* Sieb. et Zucc.) (Yamane et al., 2008), almond (*P. dulcis*) (Prudencio et al., 2018), Japanese pear (*Pyrus pyrifolia* Nakai) (Saito et al., 2013), apple (*Malus* × *domestica* Borkh.) (Mimida et al., 2015) and some additional plant genera including *Euphorbia* (Horvath et al., 2010), *Actinidia* (Wu et al., 2012) and *Betula* (Elo et al., 2001).

Apricot (*P. armeniaca L.*) productivity is much affected by its early-flowering time since unfavorable environmental conditions in late winter/early spring frequently result in a considerable yield loss in many producing countries. The aim of the present study was to identify apricot *CBF* and *DAM* gene sequences that may contribute to bud dormancy regulation. Sequence homology and phylogenetic analysis were used to confirm the putative biological function of the newly identified apricot gene sequences. In addition, we used quantitative real-time PCR to follow the changes in gene expression over the developmental stages of flower buds and during two consecutive dormant periods of field-grown trees. The regulatory role of the identified genes was further tested by comparing the gene expression profiles of apricot cultivars differing in their flowering time.

## Materials and Methods

### Plant Material

Four apricot (*Prunus armeniaca* L.) cultivars were sampled in the apricot germplasm collection of the Department of Genetics and Plant Breeding, Szent István University (Budapest, Hungary) from November to March in 2015/2016 and 2016/2017. The North American cultivars, “Aurora” (syn. “Early Blush” and NJA 53) and “Goldrich” (syn. “Sun Giant”) are two economically important early-flowering cultivars, while the late-flowering North American “Stella” and Central Asian “Zard” could be potentially used in breeding programs due to favorable characteristics including resistance to devastating diseases. According to previous studies, “Aurora” and “Goldrich” show limited resistance to chilling conditions while both “Stella” and “Zard” have better frost tolerance (Szalay et al., 2006; Milatovic et al., 2013). For the isolation of the intron-less *ParCBF1* gene, genomic DNA was extracted from the leaves of an additional apricot cultivar, “Korai zamatos,” while *ParDAM5* and *ParDAM6* sequences containing several introns were amplified from cDNA obtained from ‘Zard’ flower buds. In addition, flower buds were collected from all four cultivars for gene expression analysis.

### Determination of Chilling Requirements and Flowering Dates

Hourly air temperatures were measured by a PT100 1/3 Class B temperature sensor with ± 1°C accuracy as implemented in the iMETOS^®^ IMT200 (Pessl Instruments, Weiz, Austria) automatic weather station. The instrument was located in an open area within 400 m of the orchard. The amount of cold received by the plants were quantified using the chill units of the Utah model (Richardson et al., 1974) and portions of the Dynamic model (Fishman et al., 1987). The date of breaking endodormancy was determined by forcing apricot branches with approximately 100 buds/cultivar according to the method described by Ruiz et al. (2007). Flowering date was recorded when 50% of 500 tested flower buds were open.

### DNA Extraction

The total genomic DNA was extracted from the leaves of “Korai zamatos” cultivar using the DNeasy Plant Mini Kit (QIAGEN, Hilden, Germany). The quantity and quality of DNA were analyzed by NanoDrop ND-1000 spectrophotometer (NanoDrop Technologies Inc., Wilmington, DE, United States).

### RNA Extraction and Reverse Transcription

Flower buds of the four tested apricot cultivars were collected 8 and 7 times in two consecutive dormant periods, 2015/2016 and 2016/2017, respectively, from leaf fall (November) to the beginning of bloom (mid of March). Samples have been frozen immediately in liquid nitrogen and stored at −80°C. Total RNA from approx. 100 mg of bud tissue was extracted using the protocol of Jaakola et al. (2001). After DNase I treatment (Thermo Fisher Scientific) to eliminate the possible genomic DNA contamination, approx. 2 ng of total RNA were reverse-transcribed using an oligo (dT)_20_ primer with RevertAid H Minus First Strand cDNA Synthesis Kit (Thermo Fisher Scientific) following the manufacturer’s instructions.

### Polymerase Chain Reaction (PCR) Analysis and Sequencing of PCR Products

For each sample, PCR amplification was performed in a reaction volume of 25 μL containing 20–50 ng of DNA, 10X DreamTaq Green Buffer (Thermo Fisher Scientific, Waltham, MA, United States) with final concentrations of 4.5 mM MgCl_2_, 0.2 mM of dNTPs, and 0.75 U of DreamTaq DNA Polymerase (Thermo Fisher Scientific). Specific published primers, designed for related *Prunus* species, were applied for the amplification of target sequences (Table 1). The PCR products were separated on 1% TBE agarose gels at 80 V for 30 min and DNA bands were stained with ethidium bromide. Fragment sizes were estimated by comparison with the 100-bp DNA ladder (Promega, Mannheim, Germany).

**Table 1:** Primers used for the isolation of *Prunus armeniaca CBF1* and *DAM5-6* sequences.

Species	Primer	Sequence (5′ → 3′)	References
*P. dulcis*	CBF-F	GCCCCAGTCGAGTTTGTTGTC	Barros et al., 2012b
	CBF-R	AGCATTGCGATGGAGAAAGAAG	
*P. persica*	CBF1-F	AGGGCTTCTTCTTTCTCCAC	Wisniewski et al., 2011
	CBF1-R	AAATCTTTATGTTCGACTCACTCA	
	DAM5/1-F	ATCTCCACCACCTGCAACAGT	Yamane et al., 2011a
	DAM5/1-R	CTTCTTAACGCCCCAGTTTGAG	
	DAM5/2-F	CCCCGAAACCCACCAACGAAGATG	Bielenberg et al., 2008
	DAM5/2-R	CAGCACTGTTGCAGGTGGTG	
	DAM6-F	CCAACAACCAGTTAAGGCAGAAGA	Bielenberg et al., 2008
	DAM6-R	GGAAGCCCCAGTTTGAGAGA	

PCR products were cloned into the pTZ57R/T plasmid vector using the InsTAclone PCR Cloning Kit (Thermo Fisher Scientific) and JM109 competent cells, isolated with GeneJET^TM^ Plasmid Miniprep Kit (Thermo Fisher Scientific) and sequenced using an ABI 3500 XL Genetic Analyzer (Applied Biosystems, Foster City, CA, United States).

### Gene Expression Analysis by Quantitative PCR

The expression level of *P. armeniaca CBF* and *DAM5-6* genes was determined by means of real-time PCR (ABI 7500 Fast Real-Time PCR instrument, Applied Biosystems) using PB20.17-05 qPCRBIO SyGreen Blue Mix (Nucleotest Bio Ltd., Budapest, Hungary). The relative fold change (FC) values were calculated with the ΔΔCt method (Bookout and Mangelsdorf, 2003). The quantitative PCR primers were designed in this study from specific regions of the newly identified *ParCBF1*, *ParDAM5*, and *ParDAM6* genes (Table 2).

**Table 2:** Primers designed in this study to quantify the expression of *ParCBF1*, *ParDAM5-6* and the reference gene.

Primer	Sequence (5′ → 3′)	Annealing temperature (°C)	Amplicon length (bp)
qACT-F	GTGCCTGCCATGTATGTTGCCA	60	226
qACT-R	CAGTGGTGGTGAACATGTACCCYC		
qCBF-F	GGCTACTTGAACTGGGATGACATG	60	104
qCBF-R	ACACAAACAAATACATGATTGAC		
qDAM5-F	GCTTATGGATCCGGAGAGGCTGAATA	60	101
qDAM5-R	CAGCACTGTTGCAGGTGGTGGAGATA		
qDAM6-F	GTTTGTGGAGCCGGAGACGTTGATT	60	100
qDAM6-R	GCAGCTGGTGGAGGTGGCAATTTGG		

### Evaluation of Male Gametophyte Developmental Stages

The male gametophyte development (microsporogenesis) was studied in 2016 and 2017 from January to March. Samples were collected six times in both years. For each of the four cultivars, 8–10 flower buds were studied. Harvested buds were kept in liquid nitrogen during transportation. Anthers removed from the buds, were squashed in aceto-carmine (2%) for 5 min and then analyzed using a Zeiss Axio Imager 2 (Carl Zeiss, Thornwood, NY, United States) optical microscope at 400 × magnification. Pictures were taken with a Zeiss Axio Cam digital camera.

### Bioinformatics

*Prunus armeniaca CBF* and *DAM5-6* sequences were used as query sequences for the NCBI MegaBLAST (Morgulis et al., 2008) analysis. An alignment of sequences was carried out using MEGA6 (Tamura et al., 2011) and presented using BioEdit v. 7.2.0. (Hall, 1999). Primers were designed manually and then checked using the Oligo Analyzer 3.1 software ^1^.

To demonstrate the molecular structure of the typical protein-domains of CBF and DAM transcription factors, sequence homology was used in the SWISS-MODEL server (Arnold et al., 2006) and Global Model Quality Estimates and Qualitative Model Energy Analysis were also determined as quality estimators of the models (Benkert et al., 2008).

The evolutionary history of *CBF* and *DAM* sequences was inferred using the Maximum Likelihood method based on the JTT matrix-based model (Jones et al., 1992). Initial tree(s) for the heuristic search were obtained automatically using the Neighbor-Joining and BioNJ algorithms to a matrix of pairwise distances estimated using a JTT model, and then selecting the topology with superior log likelihood value. The analysis involved 27 amino acid sequences. There were a total of 280 positions in the final dataset. The bootstrap consensus tree inferred from 1,000 heuristic replicates was taken to represent the evolutionary history of the operational taxonomic units (Felsenstein, 1985). Branches corresponding to partitions reproduced in less than 80% bootstrap replicates were not considered statistically supported clades. Evolutionary analyses were conducted using MEGA5.1 (Tamura et al., 2011).

### Statistical Analyses

Real-time quantitative PCR data presented for each sample represent the mean values determined for three independent replicates. After tested for normal distribution and equality of variances, one-way analysis of variance (ANOVA) and Duncan’s multiple range test with *P* < 0.05 was carried out to determine significant differences. Statistical analyses were carried out using SPSS 13.0 (SPSS Inc., Chicago, IL, United States).

## Results

### Identification and Characterization of *ParCBF1*

**FIGURE 1:**
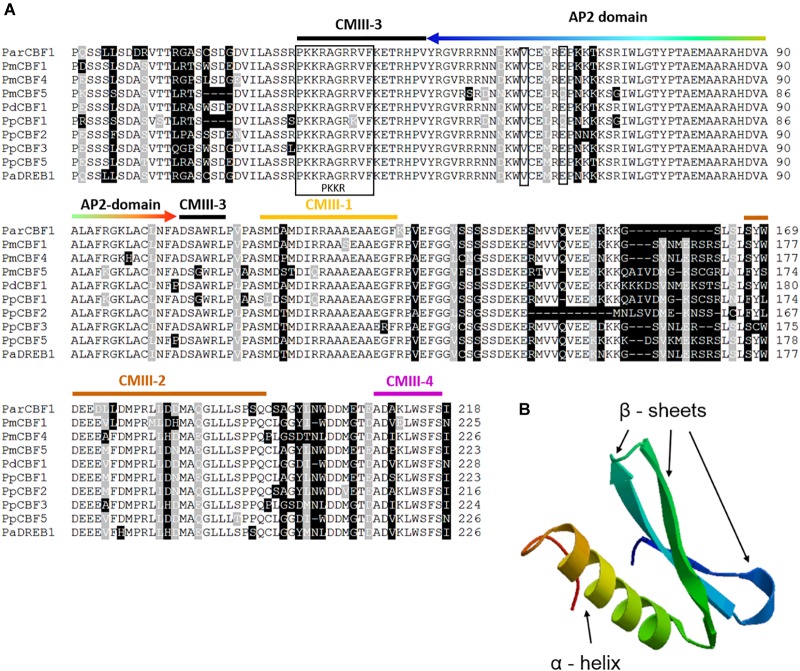
Sequence alignment of *Prunus* C-repeat Binding Factors (CBF) and the structure of *Prunus armeniaca* CBF1 protein. **(A)** Multiple alignment of the deduced amino acid sequence of *P. armeniaca* CBF and other *Prunus* CBF sequences. Dashes indicate gaps introduced to optimize the alignment. Identical amino acids and conserved substitutions are shaded black and gray, respectively. The total number of amino acids for each deduced protein is indicated at the end of each sequence. The PKKR/PAGR nuclear localization signal and the DSAWR motif are indicated by a black frame. Further CBF signature sequences are marked by orange (CMIII-1), brown (CMIII-2), black (CMIII-3) and purple (CMIII-4) colored stripes over the sequence motifs. The DNA-bindig AP2 domain is indicated by a rainbow-colored arrow (the order of colors indicate identical amino acid positions in the alignment and ribbon diagram in **B**), two narrow frames point to the two important amino acids responsible for DNA-binding specificity (a valine and a glutamic acid in the positions 14th and 19th, respectively). The CBF sequences and their GenBank accession numbers or references are as follows: *ParCBF1* (MH464453) from *P. armeniaca* L.; *PmCBF1*, *PmCBF4* and *PmCBF5* from *P. mume* published in Zhao et al., 2018; *PdCBF1* (KJ818900) is from *P. dulcis*; *PpCBF1* (HM992943), *PpCBF2* (KC543498), *PpCBF3* (KC543499), and *PpCBF5* (KC543501) from *P. persica*; and *PaDREB1* (AB121674) from *P. avium*. **(B)** Molecular model of the AP2 domain (http://swissmodel.expasy.org). AP2 domain consists of three β-sheets and an α-helix.

An apricot *CBF*-like sequence (758 bp) has been isolated from the genomic DNA of *P. armeniaca* “Korai zamatos” (the sequence was deposited into GenBank under the accession number of MH464453). A sequence homology test was performed using the NCBI MegaBLAST algorithm that detected 29 *Prunus* sequences with *E*-values of 0. The significant matches to the query included *CBF/DREB1* gene sequences. The deduced amino acid sequences were aligned with homologous sequences from several *Prunus* species available in the NCBI database (Figure 1A). The *Par*CBF1 sequence contains the highly conserved AP2 DNA-binding domain and four CMIII domains (CMIII1-4) that are also called CBF signatures. CMIII-1, 2, and 4 are located downstream of AP2 domain, while CMIII-3 includes the PKKR/PAGR and DSAWR motifs flanking the AP2 domain on the 5′ and 3′ ends, respectively. These results show that *ParCBF1* shares significant homology with other *Prunus CBF*s and has the structural motifs required for its biological function.

To further support unimpaired activity of the identified gene, the molecular structure (ribbon diagram) of its AP2-domain was modeled (Figure 1B). For AP2 molecular modeling, *Arabidopsis At*ERF1-DNA-binding domain (1gcc) was used as template, since this is the only plant protein belonging to the AP2 family with determined 3D structure (Allen et al., 1998). The sequence identity between *Par*CBF1 and *At*ERF1-DNA-binding domain was 51.7%, and the model was characterized by a GMQE value of 0.76 and a QMEAN value of −2.74, indicating major alterations are unlikely to occur in the native 3D structure of the AP2-domain in *Par*CBF1.

**FIGURE 2:**
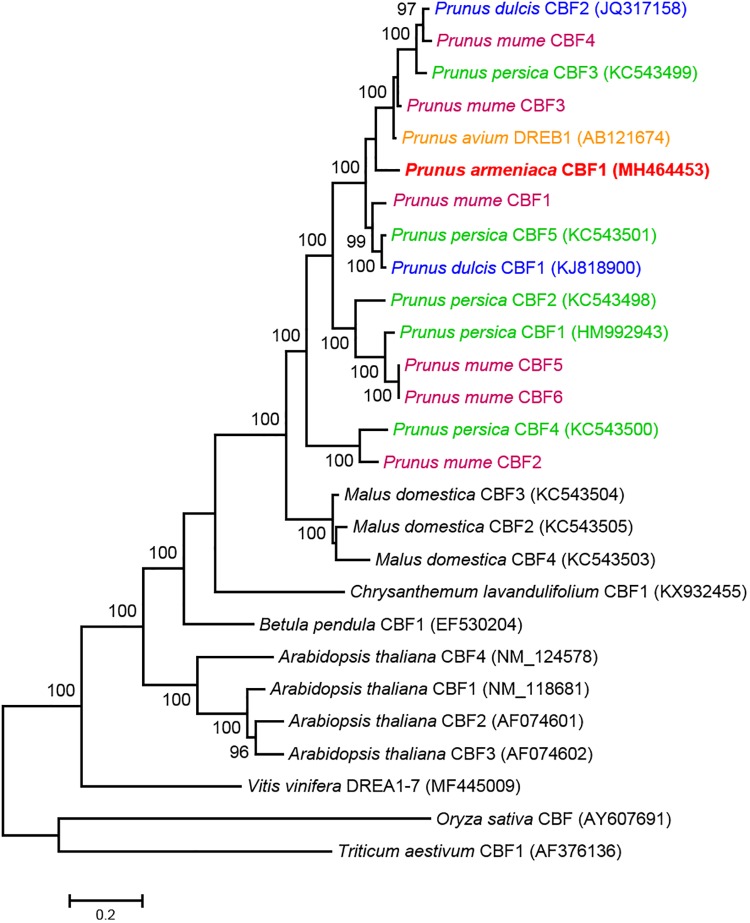
Molecular phylogenetic analysis of the amino acid sequences of *C-repeat binding factor* (*CBF*) genes using the Maximum Likelihood method. The bootstrap confidence values (%) exceeding 80% from 1000 replicates are indicated on the branches. The sequence name in red refers to the *Par*CBF1 identified in the present study. The values presented above or below the specific nodes are the percentage of replicates in which relationships were recovered. CBF sequences from different *Prunus* species are indicated by colors.

The CBF amino acid sequences previously described in monocotyledonous (*Oryza sativa* and *Triticum aestivum*) and several dicotyledonous (*Arabidopsis thaliana*, *Betula pendula*, *Vitis vinifera*, and *Malus domestica*) species were included in a phylogenetic analysis to support homology and functionality of *Par*CBF1. Most of the genes chosen for the phylogenetic analysis were described to be responsive to low temperature. The maximum likelihood tree showed that monocotyledonous sequences formed an outgroup, while each of the *Arabidopsis*, *Malus* and *Prunus* CBFs clustered together within dicotyledonous sequences and all the clusters received 100% bootstrap support (Figure 2). This pattern demonstrates a high-level similarity among *Prunus CBF* genes. The *Par*CBF1 was found within a complex sub-structured cluster that received 100% bootstrap support and included *P. dulcis*, *P. persica*, and *P. mume* CBF sequences. The sequences did not form species-specific clusters but were scattered among statistically supported (bootstrap ≥ 97%) sub-clusters and mixed with sequences from other species.

### Identification and Characterization of *ParDAM5* and *ParDAM6*

*DAM5*- (729 bp) and *DAM6*-like (230 bp) sequences were determined from cDNA obtained from flower buds of “Zard” and were deposited into GenBank under the accession numbers MH464454 and MH464455, respectively. A sequence homology test was performed using the NCBI MegaBLAST algorithm. Among the homologous sequences, *DAM5* sequences from other *Prunus* species including *P. persica*, were the most similar to *ParDAM5* with *E*-values of 0. Our *ParDAM6* sequence was significantly similar to *P. pseudocerasus*, Japanese apricot and peach sequences with *E*-values ranging from 2 × 10^−101^ to 10^−83^.

**FIGURE 3:**
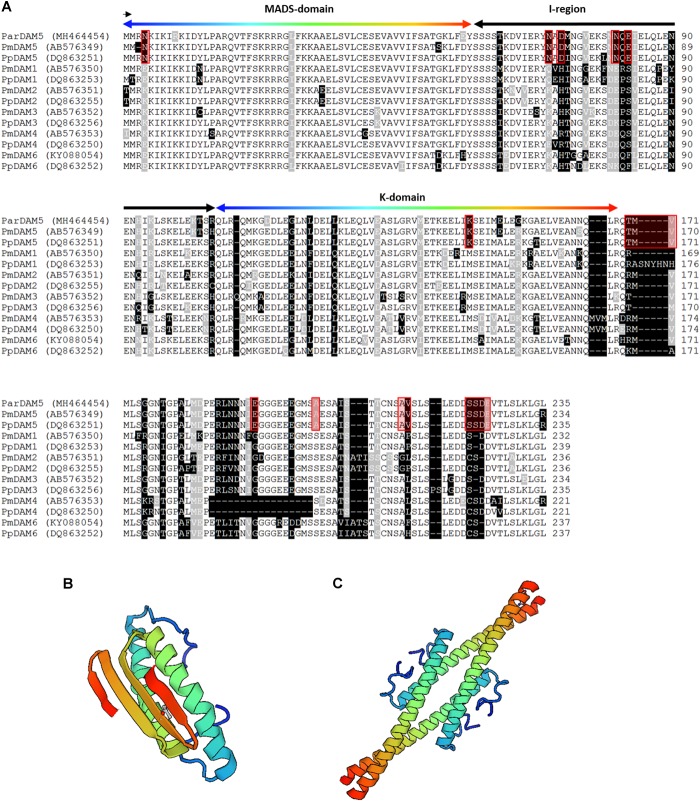
Sequence alignment of *Prunus* Dormancy-Associated MADS-BOX proteins and the structure of *Prunus armeniaca* DAM5 protein. **(A)** Multiple alignment of *P. armeniaca* DAM5 amino acid sequence with other related *Prunus* DAM sequences. Dashes indicate gaps introduced to optimize the alignment. Identical amino acids and conserved substitutions are shaded black and gray, respectively. The total number of amino acids for each deduced protein is indicated at the end of each sequence. MADS-domain, K-domain and I-region are indicated by arrows. The order of colors in the arrow over the MADS- and K-domains indicate identical amino acid positions in the alignment and ribbon diagram in **(B,C)**, respectively. Red frames indicate the amino acid positions or small motifs exclusively occurring in DAM5 sequences. **(B)** Molecular model of the MADS-domain and **(C)** the K-domain (http://swissmodel.expasy.org).

The analysis of the functional motifs gave further support for the identification of apricot sequences as *DAM5* and *DAM6* homologs. In case of *Par*DAM5, three main protein domains could have been identified in the deduced amino acid sequence: the MADS box domain (M-domain) that is the highly conserved dimerization domain at the N-terminal end, the K-domain whose function is probably to participate in protein-protein interactions, finally the variable intervening (I-) region that connects the two others (Figure 3A). The molecular structures of the MADS- and keratin-like (K-) domains are demonstrated in Figure 3B,C. For MADS-domain molecular modeling, the human MEF2 (myocyte enhancer factor 2) proteins (1c7u) was used as template, as the 3D structure of plant homologs are currently not available. The MADS domain of MEF2 is responsible for DNA-binding (Huang et al., 2000) and sequence identity between human MEF2 domain and the MADS-domain of *Par*DAM5 was 53.6% The reliability of the model was characterized by 0.76 and −2.68 GMQE and QMEAN values, respectively. SEPALLATA 3 MADS transcription factor of *Arabidopsis* (Puranik et al., 2014) was used as template for K-domain and its homo-tetramer model is characterized by GMQE and QMEAN values of 0.73 and 0.14, respectively. Although the length of the cloned *ParDAM6* sequence did not allow a similar structural analysis, many characteristic amino acid positions and motifs were detected in the deduced amino acid sequence of *Par*DAM6 that are exclusively shared with DAM6 sequences from other *Prunus* species (Supplementary Figure S3).

**FIGURE 4:**
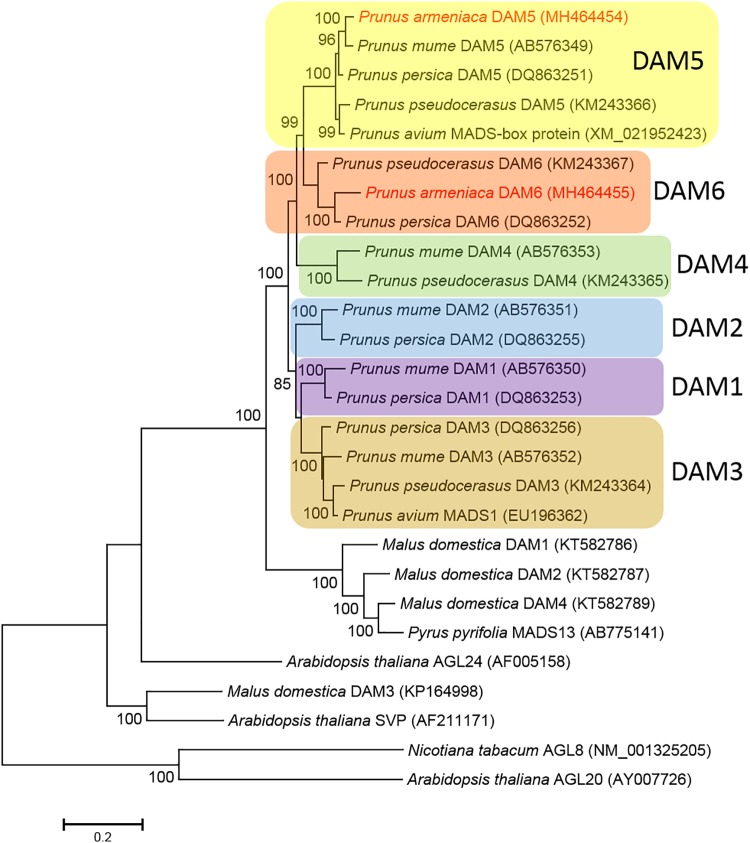
Molecular phylogenetic analysis of the deduced amino acid sequences of *dormancy-associated MADS-BOX* (*DAM*) genes using the Maximum Likelihood method. The bootstrap confidence values (%) exceeding 80% from 1000 replicates are indicated on the branches. The sequence names in red refer to *Par*DAM5 and 6 identified in the present study. The values presented above specific nodes are the percentage of replicates in which relationships were recovered. Colors indicate different *DAM* genes.

The phylogenetic analysis of DAM predicted protein sequences indicated that *Prunus* and *Malus* DAM sequences formed a sister group to *Arabidopsis thaliana* AGL24. *Malus* and *Pyrus* sequences formed a sister group to the clade encompassing sequences from all analyzed *Prunus* species. The *Prunus* clade received a 100% bootstrap support and was divided into six sub-clades according to the six different (*DAM1* to *DAM6*) genes. Each of the gene-specific sub-clades also received 100% bootstrap support. *Prunus* DAM5 and DAM6 sequences were clustered within the respective sequences from other *Prunus* species. The *Par*DAM5 sequence showed the closest relationship with *P. mume* DAM5, while *Par*DAM6 clustered with the corresponding *P. persica* sequence, with both groups receiving a 100% bootstrap support (Figure 4). Our analysis confirmed that *ParDAM5* and *ParDAM6* are evolutionarily related to *DAM5* and *DAM6* genes in other *Prunus* species, pointing to a putatively identical function in *P. armeniaca*.

### Chilling Accumulation and Anther Meiosis Time in Two Consecutive Seasons

The chilling accumulation under field conditions were followed over two consecutive dormant seasons using the chill units (CU) according to the Utah model and portions of the Dynamic model. Supplementary Figures S1, S2 show the chilling accumulation from 3 October to 30 March according to the Utah and Dynamic models, respectively. In the first part of the dormant season, the chilling accumulation was more intensive in 2016/2017 compared to 2015/2016. From 21 December, the amount of accumulated CU was higher in 2015/2016 than in 2016/2017. From 21 December to 9 January, the difference in CU between seasons was small (within 25 CU). In terms of chill portions, 2016/2017 had higher values than the preceding season only after 26 January. However, toward the end of the dormant seasons, considerably more (approx. 500 CU) and cill portions (10) accumulated in 2015/2016 than in 2016/2017.

The tendency of portion accumulation over the two dormant seasons was very similar to that shown in case of CU accumulation but results were more homogenous between dormant seasons. The variation coefficient for the chilling accumulation was smaller with the Dynamic model compared to the value of the Utah model. The variation of coefficient (cv) for the chilling accumulation until 1 March was 19.7% with the Utah model and 4.6% with the Dynamic model.

The chilling requirements (CR) for breaking dormancy are shown in Table 3 for all four apricot cultivars and in both seasons. The two early-flowering cultivars, “Aurora” and “Goldrich” had lower CR compared to the late-flowering “Stella” and “Zard.” The difference between the CR of early- and late flowering cultivars ranged between 122 and 224 CU and 7-11 portions. All cultivars were characterized by smaller CR in 2016/2017 evaluated by the Utah model, the differences between years ranged from 8.4 to 11.8%. However, the Dynamic model provided more homogenous results with variations between years equal to or less than 1.4%. The flowering of “Aurora” and “Goldrich” occurred on the same day in both seasons, while the flowering of “Stella” and “Zard” was delayed by 1 day in 2016/2017 compared to 2015/2016.

**Table 3:** Chilling requirements of the studied apricot cultivars for breaking dormancy in two consecutive dormant periods (2015/2016 and (2016/2017).

Cultivar	Season	Breaking dormancy	Chill units (Utah model)			Portions (Dynamic model)			Flowering time
			Value	Mean	cv (%)	Value	Mean	cv (%)	
Aurora	2015/2016	January 31	1203	1136	8.4	70	70	1.4	March 23
	2016/2017	February 02	1068			69			March 23
									
Goldrich	2015/2016	February 05	1299	1213	10.1	74	74	1.4	March 24
	2016/2017	February 06	1127			73			March 24
									
Stella	2015/2016	February 13	1439	1335	11.0	80	81	1.2	March 30
	2016/2017	February 21	1231			81			March 31
									
Zard	2015/2016	February 15	1473	1360	11.8	81	81	0.0	April 01
	2016/2017	February 22	1247			81			April 02

**FIGURE 5:**
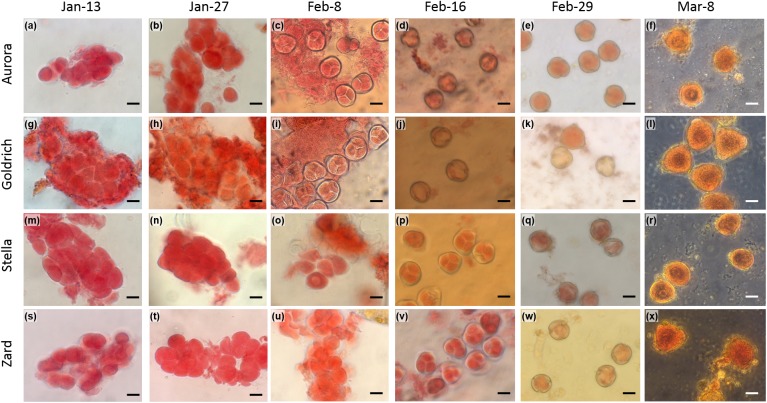
Light microscopy images of the male gametophyte development in apricot (*Prunus armeniaca*) flower buds. Two early-flowering cultivars, “Aurora” **(a–f)** and “Goldrich” **(g–l)** and two late-flowering cultivars, “Stella” **(m–r)** and “Zard” **(s–x)** were sampled in 2016 from January to March. Scale bars correspond to 20 μm.

**FIGURE 6:**
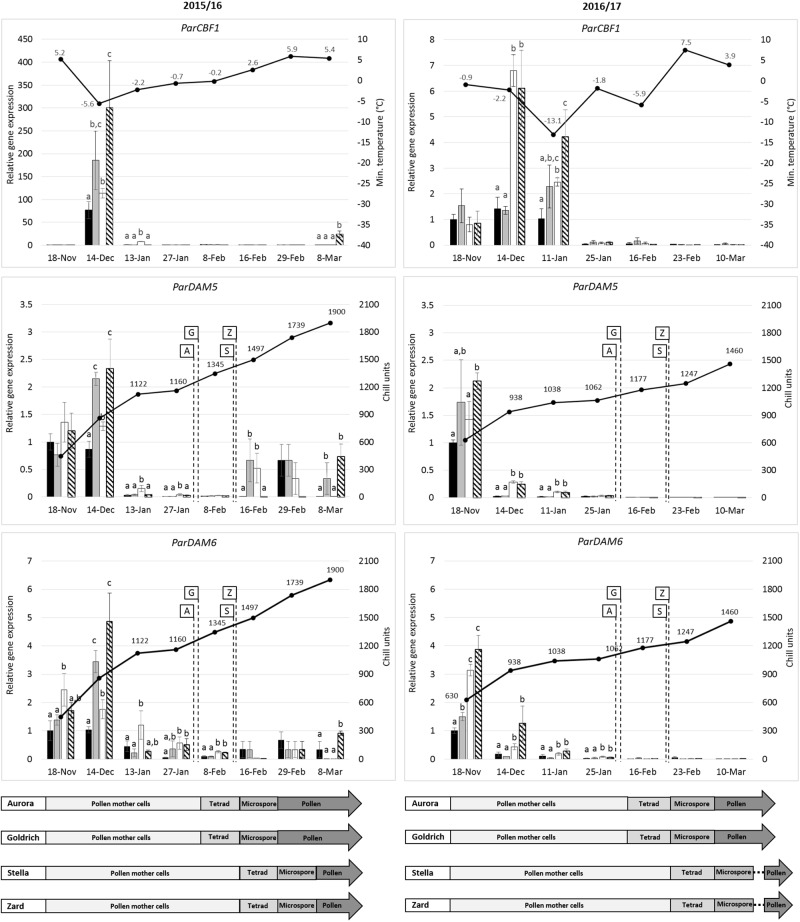
Seasonal gene expression patterns of *ParCBF1*, *ParDAM5* and *ParDAM6* in apricot (*Prunus armeniaca*) flower buds. Two early-flowering cultivars, “Aurora” (black) and “Goldrich” (gray) and two late-flowering cultivars, “Stella” (white) and “Zard” (hatched) were sampled in two consecutive dormant seasons. Real-time PCR data are means of three replicates, means followed by the same letters were not significantly different at *P* < 0.05 according to a Duncan’s multiple range test. Minimum temperatures or accumulating chill units determined for each date are indicated as black spots. An interpretative diagram shows anther developmental stages of the four assayed cultivars over sample collection dates.

We determined the anther developmental changes over the two tested dormant seasons since chilling temperatures contribute to flower bud formation in dormant trees. Figure 5 shows anther developmental stages in 2016. Four stages of microsporogenesis were distinguished in both years: archesporium – undifferentiated sporogenic tissue; premeiotic conditions (development and separation of pollen mother cells); tetrad (after meiosis) and microspores (development of pollen grains). In 2016, development and separation of pollen-mother cells started from the beginning of January, tetrad stage could be first observed at the beginning of February, the microspores could have been detected from mid-February and matured pollen grains occurred in the end of February. In 2017, the whole process was delayed and the earliest time of tetrad stage was observed in the middle of February 2017 (Figure 6). This one-week delay in male gametophyte development could have been caused by low temperatures at the beginning of February (Supplementary Table S1).

The late-flowering cultivars, “Stella” and “Zard” showed one-week delay in the timing of meiosis and the development of pollen grains in both years compared to the respective developmental stages of the early-flowering “Aurora” and “Goldrich.” The difference in timing of the anther development of early- and late flowering cultivars coincided with the fulfillment of the CR of apricot cultivars. All cultivars reached the tetrad stage after their CR was fulfilled.

### Expression Analysis of *ParCBF1* and *ParDAM5-6* Genes

Figure 6 shows the changes in relative transcript levels of the *ParCBF1* and *ParDAM5-6* genes in apricot flower buds compared to the daily minimum temperature values and chilling units, respectively, during two consecutive dormant periods (2015/2016 and 2016/2017). During the dormant period of 2015/2016, the relative expression level of *ParCBF1* was the highest in December in case of all cultivars. The transcript abundance was 4-fold higher in “Zard” flower buds than in case of “Aurora,” the most frost-sensitive genotype among the four cultivars tested in this study. In 2015 December, the increase in *ParCBF1* expression level was much higher compared to 2016/2017. Regardless of the discrepancy in the extent of increase in expression rate, the increase of *ParCBF1* expression in December coincided with temperatures falling below the freezing point in both seasons (−5.6°C in 2015 and −2.2°C in 2016). In addition, statistically significant differences were observed in *ParCBF1* expression of “Aurora” and both “Stella” and “Zard” in each of the seasons. In December 2015, “Goldrich” was also characterized by significantly higher expression rates than “Aurora.” The expression of *ParCBF1* was downregulated in all cultivars to the end of January in both seasons and remained almost zero until the time of budbreak.

A characteristic seasonal pattern was observed in the expression rate of *ParDAM5-6* genes, which were quite similar in both seasons. Transcript levels were high at the beginning of the dormant period in both years. The highest gene expression levels were detected in December 2015 and in November 2016 for both *ParDAM5* and *ParDAM6*. Then, the expression levels of both genes started to decrease gradually.

After a considerable drop in *ParDAM5* and *ParDAM6* expression, significant differences were detected among some cultivars in both seasons. In the 2015/2016 dormant season tested, “Stella” accumulated moderate although significantly higher *ParDAM5* and *ParDAM6* transcript levels in flower bud tissues in January 2016 than other cultivars. Significant differences were found between the early and late-flowering cultivars until 08 February. Correspondences among the expression rates of *ParCBF1* and *ParDAM5-6* were evident in December 2015 and January 2017 when cultivars showing higher expression rates of *ParCBF1* also had higher transcript levels for both *ParDAM5* and *ParDAM6*. However, in the end of January *ParCBF1* expression was downregulated without significant alterations among cultivars, while “Stella” still had a higher *ParDAM5* expression level and both late-flowering cultivars had higher transcript levels for *ParDAM6*. This difference could have been observed until 08 February 2016. During the 2016/2017 dormant season, between December 2016 and January 2017, both late-flowering apricot cultivars were characterized by significantly higher *ParDAM5* and *ParDAM6* expression levels compared to the early-flowering cultivars. For *ParDAM6*, the difference also occurred on 25 January. Similar variations were observed between the *ParCBF1* expression levels of early- and late-flowering apricot cultivars until 11 January.

The expression of all three genes were definitely downregulated once the CR of the cultivars had been fulfilled. It is clearly shown by the significantly higher *ParDAM6* expression rates in “Stella” and “Zard” on 08 February 2016, which resulted in a delay of 10–14 days in dormancy breaking time compared to early-flowering cultivars. Although values were smaller, significant differences in *ParDAM6* transcript levels were also detected before the end of endodormancy.

Both *ParDAM5-6* genes were characterized by somewhat higher expression rates over February and March in 2015/2016 than 2016/2017, however, characteristic differences between the early- and late-flowering cultivars were not detected any more.

## Discussion

### Identification and Structural Verification of Dormancy-Related Genes in Apricot

Apricot is one of the earliest flowering fruit trees, a fact that makes this species susceptible to yield-loss induced by unfavorable weather conditions in early spring. The initiation and release of endodormancy was shown to be regulated by a set of genes in *Prunus* species with *CBF* and *DAM* genes playing crucial roles in this complex regulatory network (Saito et al., 2015; Zhao et al., 2018). In the present study, we identified the apricot homologs of *CBF1*, *DAM5*, and *DAM6* genes and provided *in silico* and experimental data to support their impaired structure and putative function in bud dormancy regulation.

Among stone fruit species, the *CBF* genes were first isolated in sweet cherry (Kitashiba et al., 2002) and peach (Wisniewski et al., 2011) and shown to be associated with dormancy induction and freezing tolerance. Homologous sequences were also identified in *P. cerasus* (Owens et al., 2002), *P. dulcis* (Barros et al., 2012b) and *P. mume* (Guo et al., 2014). This study was carried out to identify a *CBF* homolog in apricot, *P. armeniaca*. A 758-bp sequence of the intronless gene was amplified from the genomic DNA of “Korai zamatos” apricot cultivar using the primers designed by Barros et al. (2012b) and Wisniewski et al. (2011) from the conserved regions of *Prunus CBF* sequences and the amplicon was cloned and sequenced. The MegaBLAST analysis provided significant support of its homology with *CBF/DREB1* genes of *Prunus* species. In addition, the alignment of the deduced amino acid sequence with *P. mume*, *P. dulcis*, *P. persica* and *P avium* CBF/DREB sequences confirmed the presence of the AP2 domain and several CBF signature motifs including CMIII1-4 (Figure 1). The PKK and AP2 domains seem to affect nuclear localization of CBF transcription factors, whereas the same domains in cooperation with the C-terminal hydrophobic amino acids affect transactivation (Carlow et al., 2017). Such domains were shown to be strongly conserved in functional CBF proteins (Jaglo et al., 2001; Nakano et al., 2006) that suggest *ParCBF1* is a functionally intact homologous gene in apricot.

The 3D structure of the complex of the *Arabidopsis At*ERF1-DNA-binding domain and its target DNA was determined by NMR (Allen et al., 1998) and used for molecular modeling of *Par*CBF1. The Quality estimators, GMQE and QMEAN indicated the built model was reliable and declared the typical AP2 fold with a three-stranded beta-sheet and an alpha helix almost parallel to the beta-sheet. This topology was found to be associated with contacting eight consecutive base pairs in the major groove of DNA (Allen et al., 1998) and hence it indicates an unimpaired DNA-binding ability of *Par*CBF1. Since CBF transcription factors may bind specifically to the C-repeat/dehydration-responsive (CRT/DRE) element in the promoters of *DAM* genes, the intact AP2 domain structure indicates a possible connection between CBF and dormancy-associated MADS-box proteins.

The *Prunus* genome contains several *CBF* genes. The *in silico* analysis of genome sequences detected six *CBF* genes both in *P. persica* (Wisniewski et al., 2014) and *P. mume* (Zhao et al., 2018) genomes. According to our phylogenetic analysis, the first apricot homolog (*ParCBF1*) identified in the present study is the most likely homolog of other *Prunus CBF* sequences indicating close evolutionary relatedness and putative functional similarities. Co-clustering of *PmCBF1* and *PpCBF5* was also shown by Zhao et al. (2018) indicating a close relationship between those genes labeled by different numbers in two *Prunus* species. Considering the number of *CBF* genes in *Prunus* genome, at least five additional homologs are still likely to be identified in the apricot genome. The involvement of *ParCBF1* in dormancy regulation is supported by a similar physiological role of its almond and Japanese apricot homologs (Barros et al., 2012a; Zhao et al., 2018).

The *ParDAM5* and *6* cDNA sequences were isolated using the gene-specific primers designed by Yamane et al. (2011b) and Bielenberg et al. (2008), respectively. The homology searches indicated that they are closely related to the corresponding *Prunus* sequences in the GenBank database. All characteristic domains (including the MADS box, I region and K domain) of the type II (MIKC^C^) subfamily of MADS-box transcription factors (Horvath, 2015) were detected in the *ParDAM5* sequence.

Six *DAM* genes were identified in peach and Japanese apricot (Bielenberg et al., 2008; Sasaki et al., 2011) but only two of the six (*DAM5* and *DAM6*) genes were reported to be associated with endodormancy release in peach generative buds (Yamane et al., 2011a). Using primers specifically amplifying *DAM5* and *DAM6*, we determined the partial sequences of their *P. armeniaca* homologs. For *PpDAM5*, the homology was confirmed by MegaBlast and phylogenetic analyses, indicating that *ParDAM5* belongs to the super-clade of *SVP*/*AGL24 Arabidopsis* sequences as was previously reported for *P. mume DAM6* (Yamane et al., 2008). The identification of protein domains and the homology modeling of characteristic domains suggest its functional integrity.

Since putatively functional *DAM* sequences were available from several species, a careful screening of the aligned sequences helped circumscribe the amino acid positions or small motifs that are consequently characteristics of *DAM5* and *DAM6* genes and differentiate them from the rest of the *DAM* genes. For DAM5, 6 of the 11 diagnostic motifs located in the conserved domains (MADS, I and K), and 5 additional in the C terminal part of the protein (Figure 3). One of those flanked a region of indels, i.e., insertions or deletions downstream of the K domain. The 75 amino acids compared in DAM6 sequences also detected six specific motifs (Supplementary Figure S3). Among the 260 amino acid positions in the alignment of all six *Pp*DAMs (Bielenberg et al., 2008), 122 amino acid positions were invariable. The conserved amino acids are likely to be crucial to the biological function of the genes, however, the contribution of the newly identified gene-specific sequence variations to the physiological function of the corresponding genes might be an interesting scope for future studies.

### Functional Verification of Dormancy-Related Genes in Apricot

The newly isolated *ParCBF1* and *ParDAM5* sequences show all structural features that are indispensable for the relevant biological functions. For *ParDAM6*, only a small part of the cDNA was sequenced that allowed the quantification of gene expression levels. *CBF* and *DAM* genes were described to have characteristic expression profiles over the dormant season and dormancy release (Jiménez et al., 2010; Sasaki et al., 2011; Yamane et al., 2011a,b; Barros et al., 2012a; Zhao et al., 2018), which makes them appropriate candidates for the internal factors controlling endodormancy of temperate trees.

The expression of *ParCBF1* was associated with decreasing ambient temperatures. In 2015, the peak of *ParCBF1* expression occurred in December, the coldest month in the 2015/2016 dormant period. After that, temperature was going to be increased and *ParCBF1* expression immediately dropped. January was colder in 2017 than 2016 and the expression rate of *ParCBF1* did not show a sharp decrease compared to the year before. A rapid increase of *CBF* gene expression due to low temperature was observed in many plant species including fruit trees like apple (Wisniewski et al., 2011), almond (Barros et al., 2012a,b) and Japanese apricot (Guo et al., 2014; Zhang et al., 2013). Such a characteristic expression profile of *ParCBF1* validates its physiological role as an important element of the low temperature signaling cascade leading to dormancy induction and release in apricot.

The presence of a conserved core sequence motif (CCGAC) in the promoter region of *DAM5* and *DAM6* genes in peach and Japanese apricot suggests their participation in the CBF regulon (Yamane et al., 2011b; Zhao et al., 2018). *ParDAM5* and *ParDAM6* had similar expression patterns as their peach and Japanese apricot homologs (Yamane et al., 2011a,b; Zhao et al., 2018). Significant differences between the expression levels of *ParDAM6* in early- and late-flowering cultivars could be also detected 12–14 days later than in case of *ParDAM5*, which indicates differences in their regulation. The expression levels of *DAM*s correlated with the number of CBF-binding sites in the promoter of Japanese apricot genes with *PmDAM5* and *ParDAM6* having several sites and showing the highest levels. The expression levels of *ParDAM5* and *ParDAM6* changed according to low temperature induced *CBF1* expression rates and the fulfillment of cultivar CR. The expression levels of *ParDAM5* and *ParDAM6* were related to the daily minimum temperatures and *CBF1* expression rates: the sampling day in December 2015 was colder than in 2016 (Supplementary Table S1) and *ParCBF1* and *ParDAM5* and *6* had higher expression rates for all cultivars in 2015.

There were important differences between the climatic data of the two dormant seasons studied. In the first part of the dormant season chilling accumulation was more intensive in 2016/2017, while the second part of the dormant season was more efficient in relation to chilling accumulation in 2015/2016. The CR for breaking endodormancy was lower in 2016/2017 for all cultivars. Our results fit well with those of Viti et al. (2010) who found that in warmer dormant seasons, all tested cultivars including “Goldrich” showed lower CR than in colder years. In addition, the Dynamic model showed lower variation in chill accumulation across years that the Utah model, which indicates that Dynamic model may also provide more accurate assessment in colder climates, similarly to its reliability under warm climatic conditions (Ruiz et al., 2007; Viti et al., 2010).

The early-flowering cultivars had lower CR for endodormancy release as compared to the late-flowering cultivars in both dormant seasons. The CR of “Aurora” averaged across 2 years was similar to the 1140 ± 60 CU measured by Viti et al. (2006), while we determined slightly higher CR for “Goldrich” compared to the data collected in Spain and Italy by Viti et al. (2010). For “Stella” and “Zard,” our study provides the first CR data and they were definitely higher compared to those of the early-flowering cultivars and consistent with their late-flowering dates.

The early-flowering cultivars broke endodormancy between 31 January and 06 February. The date of endodormancy release differed in one and 2 days between the two dormant seasons for “Aurora” and “Goldrich,” respectively. Although the intensity of chilling accumulation was different in the two dormant seasons, differences practically disappeared in the second half of January that explains the nearly identical date of endodormancy release of early-flowering cultivars in both years. However, late-flowering cultivars were characterized by strikingly different endodormancy release dates in the two dormant seasons ranging from 7 (“Zard)” to 8 (“Stella”) days. It might be explained by the slower chilling accumulation in 2016/2017 resulting in a longer period for late-flowering cultivars to achieve the critical amount of chilling units in this season.

Our results indicated that early- and late-flowering cultivars broke endodormancy in the beginning and the middle of February, respectively. The expression level of *ParDAM5* and *ParDAM6* markedly decreased in the middle of January (in the season 2015/2016) or in the middle of December (2016/2017). After the downregulation of *ParDAM5* and *ParDAM6*, throughout the next 4 and 6 weeks in the seasons 2015/2016 and 2016/2017, respectively, a consequent and significant difference (*p* ≤ 0.05) occurred between the expression levels of both genes in early- (“Aurora” and “Goldrich”) and late-flowering (“Stella” and “Zard”) cultivars. Peach *PpDAM5* and *PpDAM6* were proposed to function as a dose-dependent growth inhibitor in dormant flower buds (Yamane et al., 2011a) and were characterized by lower expression rates in the low-chill peach cultivar compared to the high-chill cultivar (Yamane et al., 2011c). Our results suggest a similar function for *ParDAM5* and *ParDAM6* with higher expression rates associated with delayed endodormancy release time and consequent late-flowering time of “Stella” and “Zard” apricot cultivars.

Meiosis has been long considered a sign of endodormancy release (Szabó et al., 2002), but pollen tetrads of medium and high-chilling requirement apricot cultivars appeared earlier with respect to the end of endodormancy (Bartolini et al., 2006). In our study, the tetrad stage emerged in all four apricot cultivars after endodormancy release time (Figure 6). Our results confirm the findings of Julian et al. (2011) that winter dormancy set a boundary between the development of the sporogenous tissue and further microspore development and meiosis appears to occur around breaking of dormancy. Pollen tetrad stage occurred 7–8 days later in late-flowering apricot cultivars in both seasons. It indicates the genetically controlled mechanisms of male gametophyte development as it was also shown for other apricot cultivars (Julian et al., 2014).

In addition to the genotype-dependent difference between the assayed early- and late-flowering cultivars, timing of the anther development stages also showed a year-to-year variation with an approximate one-week delay in 2016/2017 compared to 2015/2016. Such a difference occurred in both early- and late-flowering cultivars although the flowering time was delayed by only a single day in 2017. Since chilling accumulation was more intensive in 2015/2016 after 15 January, all cultivars achieved earlier the required amount of chilling for breaking endodormancy than during the 2016/2017 dormant season. Similar differences between cold and warm winter years were also recorded for Mediterranean apricot cultivars (Julian et al., 2014). Compared to a cold winter year, cultivars with medium or high CR showed a considerable delay in reaching the tetrad stage in a warm winter year due to CR fulfilled later in the dormant period. Although flowering time was shown to be primarily influenced by the specific CR of apricot cultivars, other factors (differences in pre-blossom temperatures, irradiation etc.) may also influence flowering dates (Rodrigo and Herrero, 2002; Ruiz et al., 2007; Julian et al., 2014).

The timing of male gametophyte development in apricot flower buds is influenced by temperature to help avoid floral organ damage. The expression of the newly identified *ParCBF1* was mainly induced by low temperature, while decrease of the expression of *ParDAM5* and *ParDAM6* apricot genes coincided with the end of endodormancy stage. The difference in *ParDAM5* and *ParDAM6* gene expression toward the end of endodormancy showed a clear association with the differing dates of endodormancy release and consequent flowering times in case of cultivars with differing CR. In Hungary, annual temperature has increased 0.8°C over the past century (Lakatos et al., 2011) and this warming tendency resulted in a shift of endodormancy release time by 19–23 days earlier over 24 years while flowering time occurred 3 days earlier (Szalay et al., 2019). Hence, the identification and characterization of the apricot homolog of three genes that have been associated with the onset and release of flower bud dormancy in other *Prunus* species are of crucial importance. *ParCBF1*, *ParDAM5*, and *ParDAM6* show all typical structural features and genetically and environmentally controlled expression levels over the endodormancy stages to be important elements of the molecular network behind bud dormancy of apricot trees.

## Author Contributions

JH, GG, and AH designed the study. EB and AH wrote the manuscript. All authors performed the research and critically revised the manuscript.

## Conflict of Interest Statement

The authors declare that the research was conducted in the absence of any commercial or financial relationships that could be construed as a potential conflict of interest.

## Footnotes

1 www.idtdna.com

## Supplementary Material

FIGURE S1 The accumulation of chilling units (Utah model) over the 2015/16 (black) and 2016/16 (gray) dormant periods.

FIGURE S2 The accumulation of chilling portions (Dynamic model) over the 2015/16 (black) and 2016/16 (gray) dormant periods.

FIGURE S3 Multiple sequence alignment of partial *P. armeniaca* DAM6 amino acid sequence with other related *Prunus* DAM6 sequences. Non-conservative and conservative amino acid replacements are shaded black and gray, respectively. The total number of amino acids for each deduced protein is indicated at the end of each sequence. Blue frames indicate the amino acid positions or small motifs exclusively occurring in DAM6 sequences.

TABLE S1 Daily minimum temperatures throughout the sample collection periods of the 2015/16 and 2016/17 dormant seasons.

## Abbreviations

AP2/ERF: Apetala2-Ethylene Responsive Factor
CBF: C-repeat Binding Factor
DREB1: Dehydration-Responsive Element Binding
GMQE: Global Model Quality Estimates
JTT: Jones–Taylor–Thornton Model
QMEAN: Qualitative Model Energy Analysis.
