# Supplementary figures and images for: Identification, Structural and Functional Characterization of Dormancy Regulator Genes in Apricot (Prunus armeniaca L.)
Source: Front Plant Sci. 2019 Apr 5;10:402. doi: 10.3389/fpls.2019.00402 (PMC6460505; doi:10.3389/fpls.2019.00402)

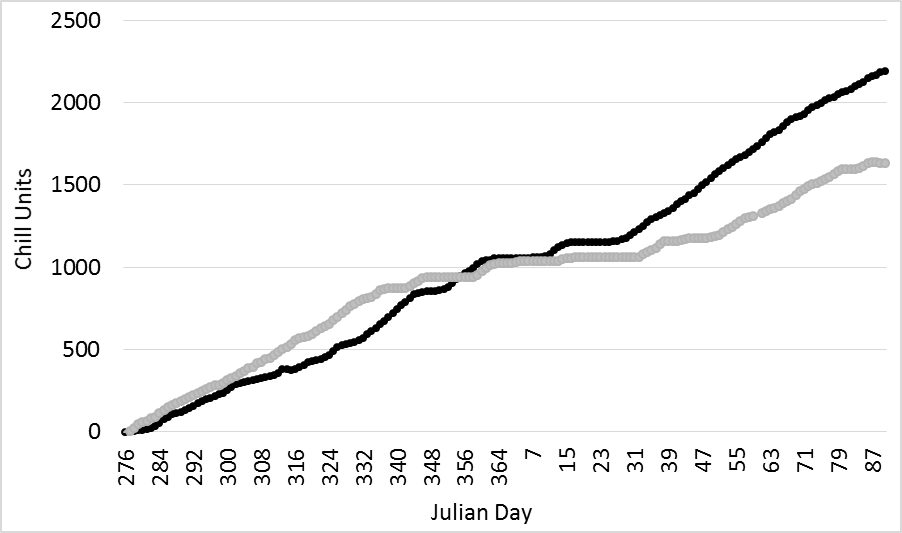

Supplement: FIGURE S1 — The accumulation of chilling units (Utah model) over the 2015/16 (black) and 2016/16 (gray) dormant periods. [file Image_1.TIF]

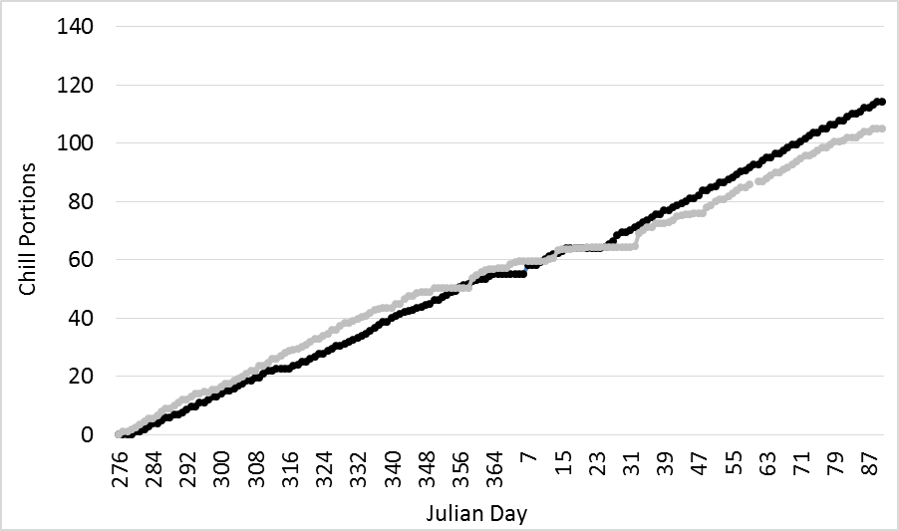

Supplement: FIGURE S2 — The accumulation of chilling portions (Dynamic model) over the 2015/16 (black) and 2016/16 (gray) dormant periods. [file Image_2.TIF]

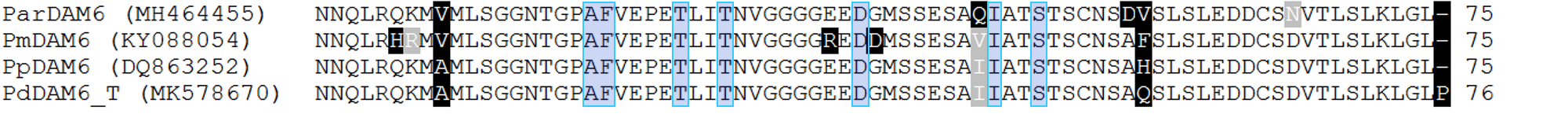

Supplement: FIGURE S3 — Multiple sequence alignment of partial P. armeniaca DAM6 amino acid sequence with other related Prunus DAM6 sequences. Non-conservative and conservative amino acid replacements are shaded black and gray, respectively. The total number of amino acids for each deduced protein is indicated at the end of each sequence. Blue frames indicate the amino acid positions or small motifs exclusively occurring in DAM6 sequences. [file Image_3.tif]
